# Clinicopathological Spectrum of Nevus Lipomatosus Superficialis: Insights From a Single-Center Study

**DOI:** 10.7759/cureus.102241

**Published:** 2026-01-25

**Authors:** Alexandra K Mawlong, Akanksha Agrawal, Lavleen Singh, Sonal Sharma

**Affiliations:** 1 Pathology, North Eastern Indira Gandhi Regional Institute of Health and Medical Sciences, Shillong, IND; 2 Pathology, Shri Dharmasthala Manjunatheshwara (SDM) College of Medical Sciences and Hospital, Dharwad, IND; 3 Pathology, All India Institute of Medical Sciences, New Delhi, New Delhi, IND; 4 Pathology, University College of Medical Sciences and Guru Teg Bahadur Hospital, New Delhi, IND

**Keywords:** classic variant, cutaneous hamartoma, dermal adipocytes, nevus lipomatosus superficialis, solitary variant

## Abstract

Introduction: Nevus lipomatosus superficialis (NLS) is a rare benign cutaneous hamartoma characterized by mature adipose tissue in the dermis. Clinically, it appears as soft, skin-colored or yellowish papules or nodules. It manifests in classical or solitary form, often misdiagnosed as lipofibroma, acrochordon, or papillomas. NLS is frequently misdiagnosed because its clinical and cytological features overlap with those of subcutaneous lipomatous lesions, while its defining characteristic (ectopic adipocytes within the dermis) is only evident on histopathological examination. This study describes the clinico-histopathological features of the solitary variant of NLS to minimize diagnostic inaccuracy.

Methods: Fifteen histopathologically confirmed NLS cases were studied over two years, with clinical presentations and morphology of lesions systematically analyzed and tabulated.

Results: A strong female preponderance was noted (male-to-female ratio = 1:14), with a mean age of 39.5 years. The thigh (five cases, 33.3%) and buttocks (four cases, 26.7%) were the most common sites, followed by the back (two cases, 13.3%) and the nape, axilla, inguinal region, and shoulder (each 6.7%). All lesions were of the solitary variant. Histology showed epidermal atrophy and mature adipocytic dermal lobules mixed with collagen. Clinically, all cases were misdiagnosed, with NLS being confirmed only after histopathological examination.

Conclusion: NLS is a rare cutaneous malformation. In this study, all histopathologically diagnosed NLS cases were of the solitary variant with a female predominance. However, larger studies are needed to confirm these patterns. Accurate clinical and histopathological recognition is crucial to avoid misdiagnosis with other common benign skin lesions.

## Introduction

Benign and malignant lipomatous tumors are the most common neoplasms of subcutaneous and deep soft tissues in adults. In contrast, purely cutaneous lipogenic neoplasms are exceptionally rare. Some dermal lipogenic neoplasms are characterized by specific clinicopathologic features in comparison with more deeply located tumors [1]. First described by Hoffman and Zurhelle in 1921, nevus lipomatosus superficialis (NLS) is rare and considered to be a developmental malformation that is characterized by ectopic adipose tissue within the upper half of the dermis [2,3]. NLS is frequently misdiagnosed because its clinical and cytological features overlap with those of subcutaneous lipomatous lesions, while its defining characteristic (ectopic adipocytes within the dermis) is only evident on histopathological examination. The etiology essentially remains unknown. Our knowledge about NLS is currently limited to small case series and case reports. Herein, we present the clinicopathological profiles of 15 cases of the solitary variant of NLS collected over two years, with the hope that this study will further explore and build upon the current body of knowledge on this entity.

## Materials and methods

Study design

This was a retrospective descriptive observational study. The data were extracted from the medical paper records at University College of Medical Sciences, New Delhi. The files of the patients were reviewed from January 2021 to December 2022. The primary objective of this study was to describe the histopathological features of NLS and to analyze its clinical spectrum in our tertiary care center.

The data collection form comprised demographic data and clinical examination findings from the medical records. Anonymized data in this study were collected from the medical records; therefore, the need for informed patient consent was not required.

Ethical considerations

Formal institutional review board (IRB) approval was waived in accordance with institutional policy for retrospective, anonymized studies. These specimens were obtained during standard clinical care, and all patients had provided informed consent at the time of biopsy for both the procedure and the accompanying histopathological examination performed for diagnostic purposes. No additional tissue was collected for research, no further patient contact occurred, and no identifiable patient information was accessed or recorded for the purposes of this study.

Inclusion criteria

Inclusion criteria included individuals who fulfilled the diagnostic criteria for NLS with biopsies demonstrating the following: (i) mature ectopic adipocytes located within the papillary dermis; (ii) the ectopic adipocytes within the papillary dermis show no continuity with subcutaneous fat; (iii) epidermis showing normal or mild acanthosis with or without hyperkeratosis; (iv) absence of cellular atypia.

Exclusion criteria

Exclusion criteria included individuals with histopathological features not consistent with NLS: (i) adipocytes present only as extension/herniation from subcutaneous fat; (ii) absence of mature adipocytes in the dermis; (iii) presence of nevomelanocytic nests, atypical cells, or other features suggesting lipoma or lipomatous hamartoma/focal dermal hypoplasia (Goltz syndrome)/nevus sebaceus/connective tissue nevus/fibroepithelial polyp with fat herniation.

Statistical analysis

Data were analyzed using descriptive statistics. Categorical variables were summarized as frequencies and percentages, and continuous variables as mean ± standard deviation or median (range), as appropriate.

## Results

We included 15 patients (one male and 14 females) who received a diagnosis of NLS on histopathology. All patients were adults varying in age from 18 to 68 years. Furthermore, descriptive analysis demonstrated an observed predominance of female patients with a male-to-female ratio of 1:14. The duration of the lesions varied from six months to 30 years, and they ranged in size from 1 x 1 cm to 10 x 7 cm. The most common site was the thighs (five cases, 33.3%) and buttocks (four cases, 26.7%), followed by the back (two cases, 13.3%) and nape of the neck, axilla, inguinal region, and shoulder (one case each, 6.7%). In this study, only the solitary form was found. Clinically, eight cases (53.3%) were misdiagnosed as papillomas, five (33.3%) as acrochordons, and one case (6.7%) each as a sebaceous cyst and soft tissue swelling. Their clinicopathological characteristics are summarized in Table 1.

**Table 1:** Clinicopathological profile of the patients.

Case	Age (years)	Sex	Site	Duration	Size (cm)	Gross appearance	Clinical diagnosis	Histopathological findings
1	44	F	Thigh	1 year	2x3	Skin-covered nodule	Papilloma	Mature adipocytes in the papillary dermis
2	22	F	Back	7 months	1.5x1.5	Skin-covered polyp with a stalk	Papilloma	Mature adipocytes in the papillary dermis
3	19	F	Buttock	5 years	4x3	Skin-covered polyp with a stalk	Papilloma	Mature adipocytes in the papillary dermis
4	26	F	Buttock	6 months	2x2	Skin-covered nodule	Sebaceous cyst	Mature adipocytes in the papillary dermis
5	61	F	Nape of the neck	1 year	1x1	Skin-covered nodule	Papilloma	Mature adipocytes in the papillary dermis
6	49	F	Buttock	1 year	3.3x3	Skin-covered nodule	Acrochordon	Mature adipocytes in the papillary dermis
7	29	F	Thigh	1 year	3x2.4	Skin-covered polyp with a stalk	Papilloma	Mature adipocytes in the papillary dermis
8	50	F	Shoulder	2 years	3x3	Skin-covered nodule	Papilloma	Mature adipocytes in the papillary dermis
9	32	F	Thigh	2 years	2x2	Skin-covered polyp with a stalk	Acrochordon	Mature adipocytes in the papillary dermis
10	18	F	Buttock	1 year	2.5x1.8	Skin-covered polyp with a stalk	Papilloma	Mature adipocytes in the papillary dermis
11	33	F	Inguinal region	2 years	5x5	Skin-covered polyp with a stalk	Acrochordon	Mature adipocytes in the papillary dermis
12	32	F	Back	1 year	1.4x1.4	Skin-covered nodule	Papilloma	Mature adipocytes in the papillary dermis
13	64	F	Thigh	10 years	4x4	Skin-covered polyp with a stalk	Acrochordon	Mature adipocytes in the papillary dermis
14	45	F	Axilla	2 years	3.8x3	Skin-covered polyp with a stalk	Acrochordon	Mature adipocytes in the papillary dermis
15	68	M	Thigh	30-35 years	10x7	Skin-covered polyp with a stalk	Soft tissue swelling	Mature adipocytes in the papillary dermis

**Figure 1:**
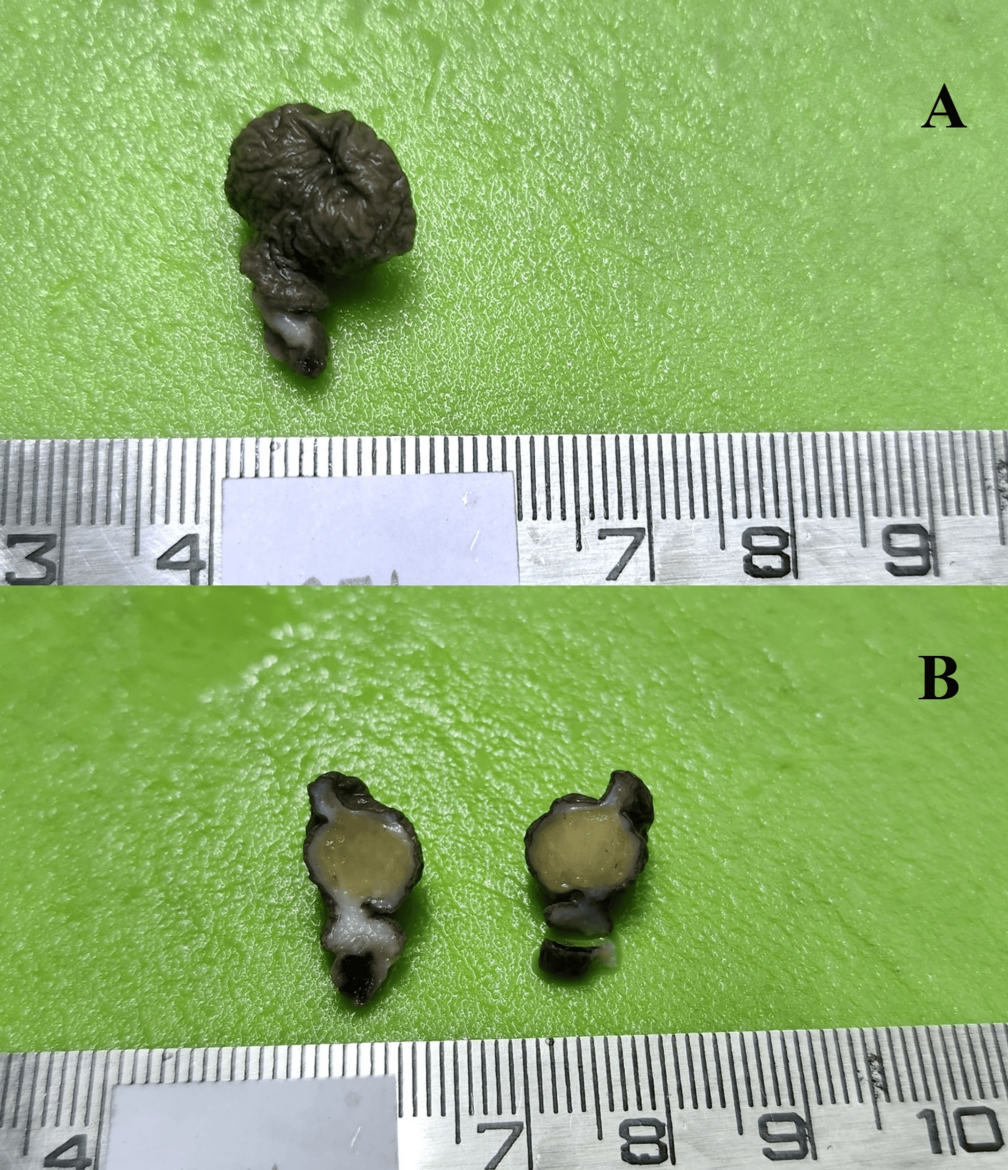
A 32-year-old female with left thigh soft tissue swelling for two years. (A) A 2 x 2 cm skin-covered polyp with a stalk. (B) A cut section revealed a homogenous cut surface, with color and consistency of normal adipose tissue.

The lesions were asymptomatic, appearing as discrete, pedunculated lesions (60%, nine cases) or as soft tissue nodules (40%, six cases) (Figure 1).

**Figure 2:**
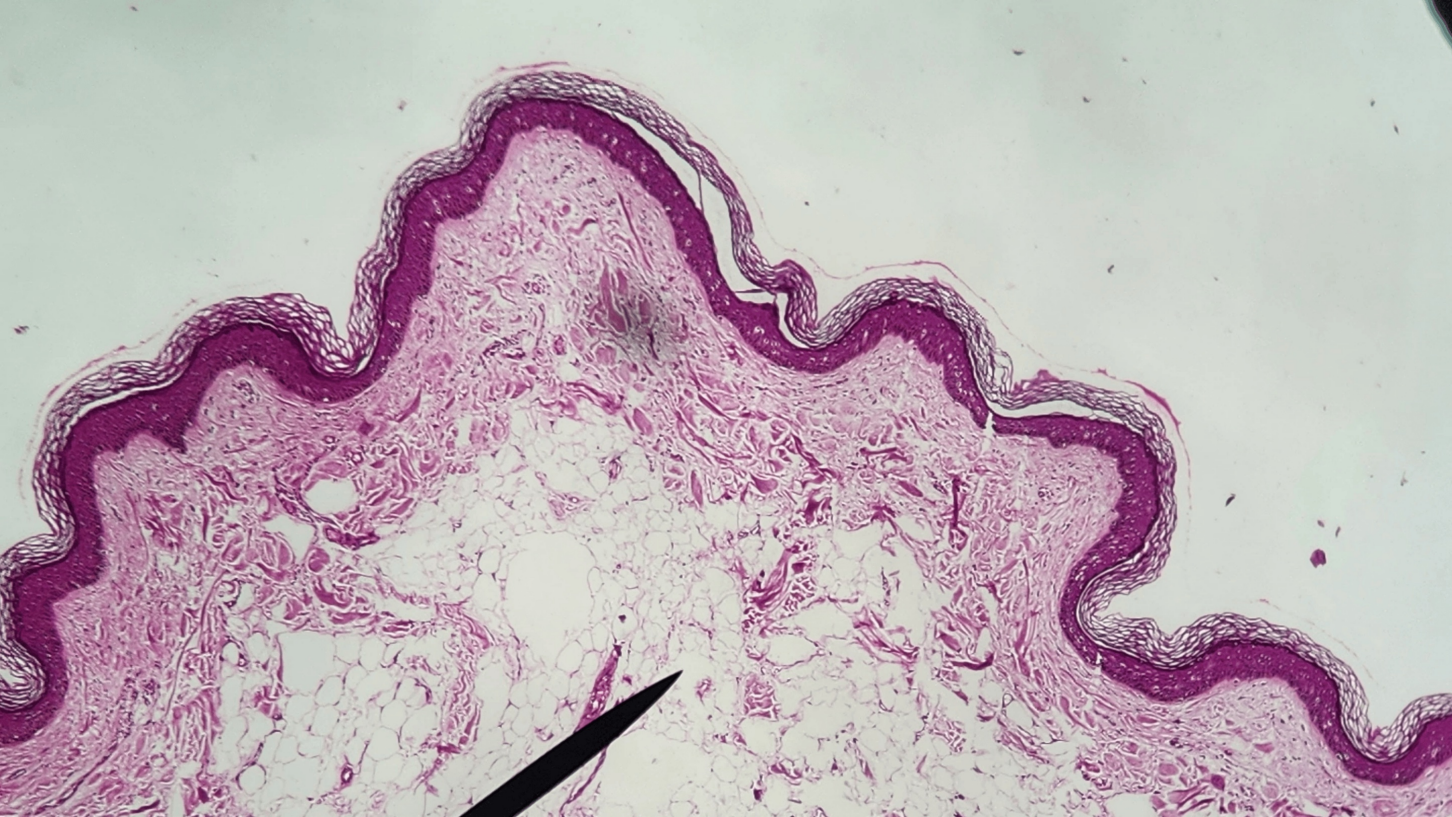
Microphotograph showing normal epidermis with lobules of mature fat cells (black pointer) embedded among collagen bundles of the dermis (H&E, 10x).

Two patients reported a progressive increase in size. On histopathological examination, the dermis showed mature adipocytes in clusters with interspersed fibrous tissue and haphazard and thickened collagen bundles. There was an increased density of proliferating capillaries within the adipocytic tissue and a reduced number of adnexal structures in the dermis. No encapsulation was seen around the lobules of mature adipocytic clusters, and there was no connection with the underlying subcutaneous fat. The epidermis showed non-specific features like hyperkeratosis, mild acanthosis, or atrophy along with increased basal cell layer pigmentation (Figure 2).

The amount of ectopic fat varied across cases. All patients were treated with surgical excision of the tumor.

## Discussion

NLS is a rare developmental anomaly characterized by ectopic adipose tissue in the upper half of the dermis. There are two subtypes. In the classic form, lesions are present at birth or develop within the first two decades of life. They are seen to have a linear, zonal distribution along natural cleavage lines of the skin and show a predilection for the pelvic girdle, sacral, and lumbar regions [4]. The second form of NLS has been reported in a wider age group, ranging from birth to 88 years and presenting clinically as a solitary, skin-colored papule or nodules with no apparent specific distribution, having been reported outside the pelvic girdle, including in the head and neck, upper extremities, distal lower extremities, and clitoris [4-9]. Jones et al. presented one of the largest series of 20 patients, in which seven had the classic variant, and 13 patients had the solitary variant [4]. Our observations echo the findings of Dudani et al., Alotaibi et al., and Palo et al. [10-12], where the solitary form was found to predominate. In contrast, Goucha et al. [13] and Kumaran et al. [14] reported the classical form to be the predominant NLS subtype. The most commonly involved sites in our study were the thighs and gluteal region, while other sites included the back, shoulder, neck, and inguinal region. Although it has been reported that NLS shows no sex predilection [4], our study revealed an observed female preponderance, with only one case out of the 15 being seen in a male. A comparative analysis of published NLS case series with the present study is compiled in Table 2.

**Table 2:** Comparative analysis of published NLS case series with the present study. * Seven other clinical diagnoses included one case each of neurofibroma, lipoma, sweat gland tumor, cobblestone nevus, connective tissue nevus, cyst, and accessory nipple. # Not mentioned in the article. NLS: nevus lipomatosus superficialis; NLCS: nevus lipomatosus cutaneous superficialis.

Author (Year of publication)	Total number of cases	Study duration	Male-to-female ratio	Type of NLS (number of cases)	Mean age at presentation in years (range)	Provisional clinical diagnosis	Commonest site
Jones et al. [4], 1975	20	14 years	1:01	Classical (7)	36 (14-64)	· Cellular nevus (5)	Buttocks
				Solitary (13)		· Papilloma (3)	
						· Warty nevus (2)	
						· NLCS (2)	
						· Acrochordon (1)	
						· Others* (7)	
Goucha et al. [13], 2011	8	14 years	3:05	Classical (7)	29 (7-41)	#	Buttocks and thighs
				Solitary (1)			
Kumaran et al. [14], 2013	8	11 years	3:01	Classical (5)	15.2 (0.3-35)	· Neurofibromatosis or collagenoma in classical forms	Lower back & gluteal region
				Solitary (2)		· Acrochordon in solitary forms	
				Classical & solitary (1)			
Dudani et al. [10], 2016	7	10 months	2:05	Solitary (7)	42.5 (32-56)	· Papilloma (4)	Thigh
						· Acrochordon (3)	
								Alotaibi et al. [11], 2018	5	5 years	1:04	Classical (1)	36 (12-58)	· NLCS (1)	Lower limbs
Solitary (4)	· Papilloma or acrochordon (4)														
		Palo et al. [12], 2022	6	20 months	1:01	Classical (1)	30.8 (18-47)					· NLCS (2)		Lower back and thigh	
						Solitary (5)						· Papilloma (3)			
· Acrochordon (1)														
Current study	15	2 years	1:14	Solitary (15)	39.4 (18-68)	· Papilloma (8)	Thigh and buttocks
						· Acrochordon (5)	
						· Sebaceous cyst (1)	
						· Soft tissue swelling (1)	

The pathogenesis of NLS remains unclear. Various hypotheses include origin of adipocytes from pericytes of dermal blood vessels, metaplasia of dermal connective tissue to adipose tissue, and developmental displacement of adipose tissue during embryonic life [12]. The single case that has been analyzed cytogenetically showed deletion of 2p24 [3].

Histopathological examination reveals groups and strands of fat embedded among collagen bundles in the dermis, as high as the papillary dermis. The amount of fatty tissue can vary from small foci around the subpapillary vessels to lesions showing relatively large amounts of fat. These fat lobules are irregularly distributed in the dermis, with the delineation between the dermis and hypodermis being ill-defined or lost. The overlying epidermis may show no changes or may be acanthotic or hyperpigmented. Myxoid changes are often seen in variable degrees, and collagen may be thickened [13]. Histopathology of the cases in our study demonstrated mature adipocytes in the dermis with no encapsulation or connection with subcutaneous fat and no distinct epidermal changes. The amount of ectopic fat was variable, with the dermis showing thickened collagen bundles and increased density of small blood vessels. No myxoid change was seen in the cases studied. Fine-needle aspiration cytology (FNAC) has a limited role in the diagnosis of NLS since the findings are suggestive of lipoma. Excision biopsy remains superior as it allows evaluation of the architectural relationship of mature adipocytes within the dermis, a defining histopathological feature of NLS that cannot be reliably assessed on FNAC [8].

None of the patients in our study had a correct preoperative clinical diagnosis. It was noted that the solitary form was commonly misdiagnosed clinically as a papilloma in most cases. Other differentials included acrochordon and sebaceous cyst. Other conditions which may be confused with NLS include nevus sebaceous, neurofibroma, lymphangioma, hemangioma, and focal dermal hypoplasia (Goltz syndrome). Histopathologically, there is minimal to no adipose tissue in the dermis in acrochordon. The diagnosis of pedunculated lipofibroma can be confirmed by the histopathologic findings showing isolated groups of ectopic mature adipocytes within the dermis, with the absence of skin appendages. Differentiation from an acrochordon with herniation of adipose tissue (lipofibroma or dermatofibroma) may not be possible and bears little clinical significance. Mature dermal melanocytic nevi and neurofibromas occasionally show adipose metaplasia. In the case of Goltz syndrome, there is an absence of collagen in the atrophic dermis, and skin appendages are absent. Artifactual spaces in the dermis can mimic fatty infiltration, a finding deemed pseudolipomatosis cutis. Fibroblastic connective tissue nevus can exhibit adipose tissue in the dermis but is limited to the reticular dermis [9,15,16].

Surgical resection is curative and usually sought for cosmetic reasons. NLS has also been associated with multiple cutaneous disorders such as follicular papules, hypertrophic pilosebaceous units, angiokeratoma of Fordyce, café-au-lait macules, scattered leukoderma, and hemangioma; however, malignant change has not been reported [12].

Limitations

This study has several limitations. Its retrospective design, small sample size, and single-center nature limit generalizability and preclude inferential statistical analysis. Potential referral and selection biases may have influenced case composition, and the cohort exclusively represents the solitary variant of NLS. In addition, the absence of standardized long-term follow-up restricts assessment of outcomes, and the descriptive nature of the data does not allow epidemiologic or incidence-based conclusions.

## Conclusions

This study highlights the need to consider NLS as a differential in the appropriate clinical setting, as evidenced by the fact that none of the patients reported in our study, as well as most of the patients in other studies, received a correct provisional clinical diagnosis. Studies describing large case series of patients are rare, with ours representing one of the largest studies of 15 patients over a short span of two years. We observed a comparatively large number of cases over a shorter duration. This finding, along with the paucity of literature from the Indian subcontinent, prompted us to present our data to increase awareness about its incidence. Post-surgical recurrence is rare, and a histopathological examination is mandatory for a final diagnosis.
